# Complex Disease Interventions from a Network Model for Type 2 Diabetes

**DOI:** 10.1371/journal.pone.0065854

**Published:** 2013-06-11

**Authors:** Deniz Rende, Nihat Baysal, Betul Kirdar

**Affiliations:** 1 Department of Materials Science and Engineering, Rensselaer Polytechnic Institute, Troy, New York, United States of America; 2 Rensselaer Nanotechnology Center, Rensselaer Polytechnic Institute, Troy, New York, United States of America; 3 Department of Chemical and Biological Engineering, Rensselaer Polytechnic Institute, Troy, New York, United States of America; 4 Department of Chemical Engineering, Bogazici University, Bebek, Istanbul, Turkey; CRCHUM-Montreal Diabetes Research Center, Canada

## Abstract

There is accumulating evidence that the proteins encoded by the genes associated with a common disorder interact with each other, participate in similar pathways and share GO terms. It has been anticipated that the functional modules in a disease related functional linkage network are informative to reveal significant metabolic processes and disease’s associations with other complex disorders. In the current study, Type 2 diabetes associated functional linkage network (T2DFN) containing 2770 proteins and 15041 linkages was constructed. The functional modules in this network were scored and evaluated in terms of shared pathways, co-localization, co-expression and associations with similar diseases. The assembly of top scoring overlapping members in the functional modules revealed that, along with the well known biological pathways, circadian rhythm, diverse actions of nuclear receptors in steroid and retinoic acid metabolisms have significant occurrence in the pathophysiology of the disease. The disease’s association with other metabolic and neuromuscular disorders was established through shared proteins. Nuclear receptor NRIP1 has a pivotal role in lipid and carbohydrate metabolism, indicating the need to investigate subsequent effects of NRIP1 on Type 2 diabetes. Our study also revealed that CREB binding protein (CREBBP) and cardiotrophin-1 (CTF1) have suggestive roles in linking Type 2 diabetes and neuromuscular diseases.

## Introduction

Systems biology approaches to diseases arise from a simple hypothesis that genes contributing to a common disorder have an increased tendency for their products to be linked at various levels of functionality, including protein-protein interaction, co-expression, co-regulation and share Gene Ontology terms [1]. Complex diseases have long been known to emerge from an impaired function of a single protein or a protein cluster that alter the general functionality. Statistically significant pathogenic overlap between the complex disorders results possibly from the variations in linked genes encoding proteins that are a part of a functional module. Hence, systems based approaches have found a wide range of applications for the identification of the putative proteins and revealing the underlying biological processes. The identification of disease-causing genes not only facilitates the understanding of the protein function that provides direct insight into the progression of the disease but also points out potential drug targets for further research.

In the last decades, significant efforts have been expanded to understand the contribution of genetic factors to the development of complex diseases with the hope that discovering these genetic factors will provide fundamental insights for pathogenesis, diagnosis and treatment [2], [3]. Most of these studies revealed the importance of underlying biological pathways and shared genes among the diseases. Thereby, systems biology based approaches emerged as powerful tools to identify of the molecular mechanisms underlying complex disorders and their relationships with other complex disorders including various types of cancers [4]–[7], cardiovascular disease [8], [9], neurological diseases [10], [11], diabetes [12], [13], asthma [14] and aging [15], [16].

Type 2 diabetes (T2D), or non-insulin dependent diabetes mellitus (NIDDM), is the most common form of the disease world-wide, accounting for over 90 per cent of diabetes cases, [17] where 336 million people worldwide now have Type 2 diabetes, and diabetes is responsible for 4.6 million deaths each year. These numbers highlights the fact that diabetes is one of the prospective pandemics. [18] Type 2 diabetes is characterized by a combination of impaired insulin secretion and insulin action, both of which precede and predict the onset of disease. Although environmental factors, such as dietary habits, obesity and sedentary life [19] play important roles in the progress of the disease, it is now well-known phenomenon that the disease susceptibility is influenced by genetic factors. Despite strenuous efforts over the last two decades had been embarked on the identification of genetic variants that contribute to individual differences in predisposition of T2D, susceptibility genes are mostly identified through genome-wide analysis [20]–[35]. Transcriptome data sets related to T2D obtained from different human tissues provided a new tool for the identification of underlying molecular mechanisms of the disease [36]–[39]. Alterations in signaling pathways including adipocytokines, insulin, protein kinase C’s, FFA, EGF, Jak-STAT, MAPK, VEGF, PPAR, P13K and Wnt were reported in the pathogenesis of the disease [18], [40]–[43]. Several network based approaches which integrate co-expressed genes with interaction networks were also developed to identify affected pathways and key regulatory pathways of T2D. Both studies employed gene expression datasets and integrated these datasets with protein interactions. The up-regulated and down-regulated genes are assembled to construct subnetworks. Liu et al. revealed insulin signaling and nuclear receptor subnetworks, Sengupta et al, displayed the relation between diabetes and kidney complications, and proposed interactions that pointed vascular function in diabetic nephropathy [12], [13]. Zelezniak et al. have integrated skeletal gene expression data sets with human metabolic network reconstructions to identify key metabolic regulatory features [44]. Despite the contribution of aforementioned cellular mechanisms to the disease has been well documented, there has been growing interest in identifying genes and processes that could trigger insulin resistance beyond these metabolic pathways and regulatory mechanisms. It has also been known that high blood glucose levels damage vessels that carry oxygen and nutrients to nerves and this damage manifests itself as numbness, insensitivity to pain and loss of balance and coordination in diabetic patients. The relationship between muscle strength, motor function and diabetes has been quantified by clinical studies. [45] However, the muscle weakness and decreased motor function in diabetes patients have received limited attention, partly because these complications are not considered as life threatening, hard to monitor and shadowed by the complications of diabetes. The relation between the neurological diseases and diabetes creates an ample incentive to employ system biology tools to reveal these links.

Functional linkage networks are relevant from a systems biology point of view; the general organization principles can be conveyed using these networks. Although, protein-protein interactions from high throughput experiments are reported and deposited in publicly available databases, functional relatedness can be achieved at any level of interaction; including physical interaction as well as co-expression, co-regulation and phenotypic behavior. Functionally related genes usually act in the form of modules of highly interacting proteins encoded by these genes. These modules are considered as building blocks of biological systems and their interactions may shed light into the complex function of the whole system. While integration of information from at various levels of interaction provides insight to biological systems, it should be noted that the origin of the interaction data and the verification of results are made through same sources, which may introduce bias to the system of interest, therefore rigorous randomization algorithms and detailed literature support are required.

The idea of community structure in networks has been applied in various research fields including social communities [46], [47], the internet [48] and ecosystems [49]. Modularization in yeast protein interaction networks received much attention for gene annotation, protein function prediction, identification of regulators and novel proteins in molecular pathways [50]–[52]. There are numerous algorithms proposed to identify dense subgraphs and functional modules [53]–[56]. These algorithms assign the proteins to individual and separate clusters and prevent the enumeration of overlapping modules. The Bron-Kerbosch algorithm [57] is a rigorous clique partitioning algorithm that aims to enumerate maximal cliques within a network. Its implementation is easy compared to some other clique enumeration algorithms [58] and it has been applied to various networks ranging from social networks to large scale proteomic networks to find overlapping cliques [59]–[62]. The algorithm assigns one protein into many clusters, which is a realistic requirement, considering the fact that one protein may participate in many biological processes. [9].

In this study, we have developed an integrative modular network approach, where genes were organized into functional modules based on the topological characteristics of the constructed network to investigate Type 2 diabetes. The aim of this work is to identify distinctive biological processes for the disease, as well as novel genes shared among metabolic and neuromuscular diseases. The proposed approach was initiated from the modular architecture of the Type 2 diabetes disease related functional linkage network. A novel computational approach was developed to evaluate the functional modules in terms of shared pathways, co-localization, co-expression and associations with similar diseases. The most informative modules were selected using a non-linear model where the parameters were estimated by genetic algorithm. The assembly of top scoring functional modules through overlapping members revealed the fundamental biological processes present in the pathophysiology of the disease. Other complex diseases that have pronounced associations with the proteins included in this assembly were linked to each other through shared proteins.

## Methods

**Figure 1:**
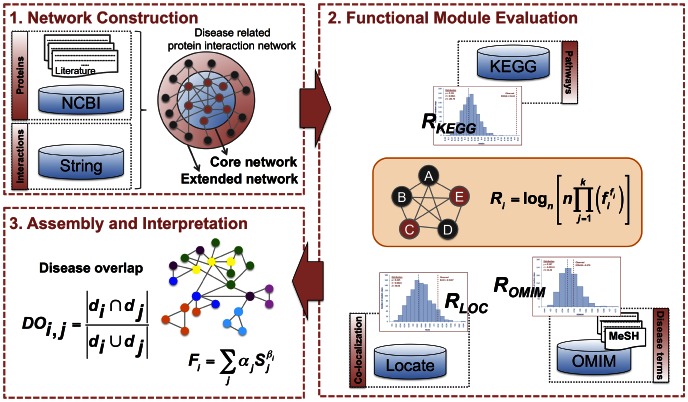
Computational framework of the study for evaluation of scoring functional modules. The databases used in the study were shown in boxes.

In this study, the functional linkage network consisting Type 2 diabetes associated proteins was constructed and analyzed in terms of modular structure. The computational framework to evaluate the functional modules enumerated from the network of interest constitutes three major stages: (i) construction of a disease related protein interaction network and its extension with neighboring proteins (ii) enumeration of functional modules, scoring these modules for co-occurring KEGG pathway terms, localization information, an integrated disease ontology composed of MeSH terms and OMIM database, co-expression patterns and evaluation of these modules with Genetic Algorithm. (iii) Assembly of the high scoring modules and calculation of disease overlapping scores. The computational framework of this study is presented in Figure 1.

### Construction of the Network

Construction of functional linkage network for Type 2 diabetes was started with 574 core proteins (c-proteins) reported to be associated with the disease in the literature [20]–[35]. Furthermore, National Center for Biotechnology Information (NCBI) database was searched for the specific disease term (Type 2 diabetes) and the resulting genes were also included.

The functional links between the proteins were extracted from STRING database v8.1 [63]. Rather than using physical evidence of protein interactions, which could be obtained through records deposited for yeast-2-hybrid experiments, the preference of linkage type was to use functionality, since modular approaches based solely on physical protein interactions generally yielded protein complexes. Hence, establishing functional linkages between proteins has been anticipated to achieve more biologically relevant structures. STRING combines available information on protein–protein interactions and assigns a confidence score according to variety of the supporting data, including physical interactions, curated biological pathway knowledge, functional linkage, co-expression profiles, as well as the co-occurrences of protein pairs in database text fields and conservation across species [63]. The core set of proteins was incorporated with the first neighbors to achieve a comprehensive disease related network constituting putative proteins that have potential associations with the disease. To select a reliable confidence score for interactions, several networks were created with different confidence scores, ranging from 900 to 990, and these networks were analyzed in terms of coverage of core set of proteins and constitution of core proteins in the network. Coverage is defined as the fraction of number of core proteins included in the network to the number of proteins initially collected. Constitution is defined as the fraction of the number of core proteins included in the network to the number of total proteins in the final network representation.

### Functional Module Identification and Evaluation

Functional modules were identified using Bron-Kerbosch (BK) algorithm [57] implemented in Python scripting language as described [9]. Functional modules were then scored using the KEGG Pathway database to associate biological pathways [64], LOCATE database to determine the co-localization information [65] and Medical Subject Headings (MeSH) [66] incorporated with OMIM database [67] to establish the links between proteins and diseases.

The consistency in a functional module was investigated by assigning a score for each category, reflecting the homogeneity of the cluster by calculating the redundancy, *R_i_*:
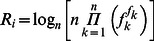
(1)where *f_k_* represents the relative frequency of the class in cluster *i* and *n* is the total number of classes in the classification scheme. These scores range between 0 and 1, where 1 indicates all the members in the functional module belong to the same classification. To assess the significance of our results, 10^3^ randomized classification schemes were generated.

After all functional modules enumerated from the network were scored according to pathway (*R_KEGG_*), localization (*R_LOC_*) and medical subject headings (*R_OMIM_*). A non-linear model was used to evaluate the functional modules with a single resulting score.
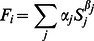
(2)where *S_j_* ∈ (log*N*, *R_KEGG_*, *R_LOC_*, *R_OMIM_*), all of which ranges between 0 and 1, 1 indicating consistency in the module; except for *N*, which denotes the size of the module. *α_j_* and *β_j_* are the nonlinear model coefficients.

Genetic Algorithm (GA) was employed to estimate the nonlinear model parameters. The nonlinear model parameters were predicted by evolving the population of tentative solutions of the model in the search space. The ten artificially generated functional modules, five of which have the highest score in each scoring scheme, were intentionally planted in the population representing the best achievable entities. The population of the modules was evolved for 100 generations. Upon the prediction of the model parameters, these model parameters were used to evaluate the functional modules and the high scoring functional modules were investigated for biological significance. (Text S1).

### Biological Processes through Non-overlapping Gene Ontology Terms

**Figure 2:**
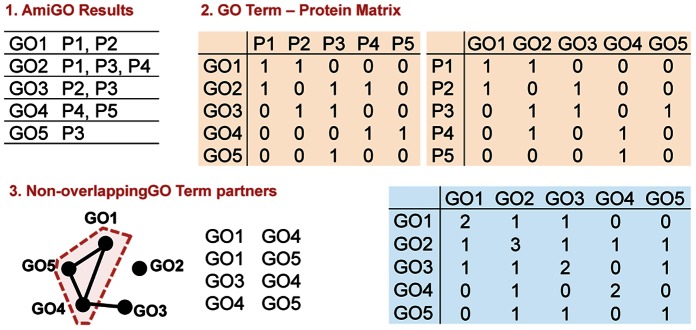
The computational framework to derive non-overlapping GO Terms.

After the functional modules were scored according to the classification schemes, the top scoring members of the modules were assembled in a condensed network. This network comprises only the proteins that are associated with each other in terms of shared pathways and localizations, co-expression and shared diseases. To determine the underlying biological processes in the condensed network, the Gene Ontology (GO) Biological Process terms associated with these proteins were extracted by AmiGO analysis (Table S3). This well-known analysis procedure yields the GO Terms, and their associated proteins as a list and comprises extensive amount of overlaps. We developed a framework to overcome these overlaps and to distinctively decipher the biological processes that are associated with the proteins in the condensed network. Figure 2 represents the schematics of the computational framework. In a typical analysis, all the proteins in the condensed network provided to the AmiGO term enrichment analysis tool, by which the GO terms associated with the proteins are listed [68]. This information was then used to construct a GO Term – protein matrix, which was then multiplied with its transpose to yield GO Term matrix. In this symmetrical matrix, the diagonal elements represent the number of proteins associated with the corresponding GO Term, non-zero elements show the number of proteins shared among two particular GO Terms and zero elements indicate the non-overlapping GO Term partners. These non-overlapping GO Terms were then listed as an interaction network. The modules with *Q* = 1 in this interaction network yields the non-overlapping GO Term groups in the condensed map. Among these configurations, the best representation of the map is conveyed through the total number of proteins assigned to GO Terms. It should be noted that, in the final configuration, non-assignment to a GO Term does not necessarily mean that a protein is not associated with a GO Term, rather it implies that this particular protein is associated with many GO Terms, therefore it cannot be included in non-overlapping GO Terms.

### Complex Disease Interventions

To associate complex diseases with each other, the proteins assembled in a condensed map and their linkages with other proteins were used. The links between the proteins and diseases were established by incorporating MeSH terms with OMIM database. All disease protein relations extracted are presented in (Table S4). For instance, the proteins in the condensed network were linked to a disease term. Hence the diseases can be associated with each other through shared proteins. Each pairwise disease association was evaluated in terms of overlapping partners by considering only the proteins in condensed in T2DFN network. A score representing the disease overlap (*DO*) was assigned to each pair of disease terms appear in the condensed network using:
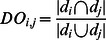
(3)where *d_i_* and *d_j_* represent the proteins associated with disease term pair. To determine the significance of our analysis, 10^3^ random control runs were performed. The proteins and randomly annotated disease terms were shuffled and overlapping scores were recalculated. The random distribution obtained for each disease term was compared with the current score. The statistical analysis was subsequently corrected by calculating FDR.

## Results and Discussion

### Construction of Type 2 Diabetes Related Functional Linkage Network

The construction of Type 2 diabetes functional linkage network was started with the proteins encoded by 574 genes (c-proteins) collected from previous studies (Table S5) [20]–[35]. The linkages between the proteins were extracted from STRING v8.1, using a selected confidence score threshold of 940 as described in materials and methods.

**Figure 3:**
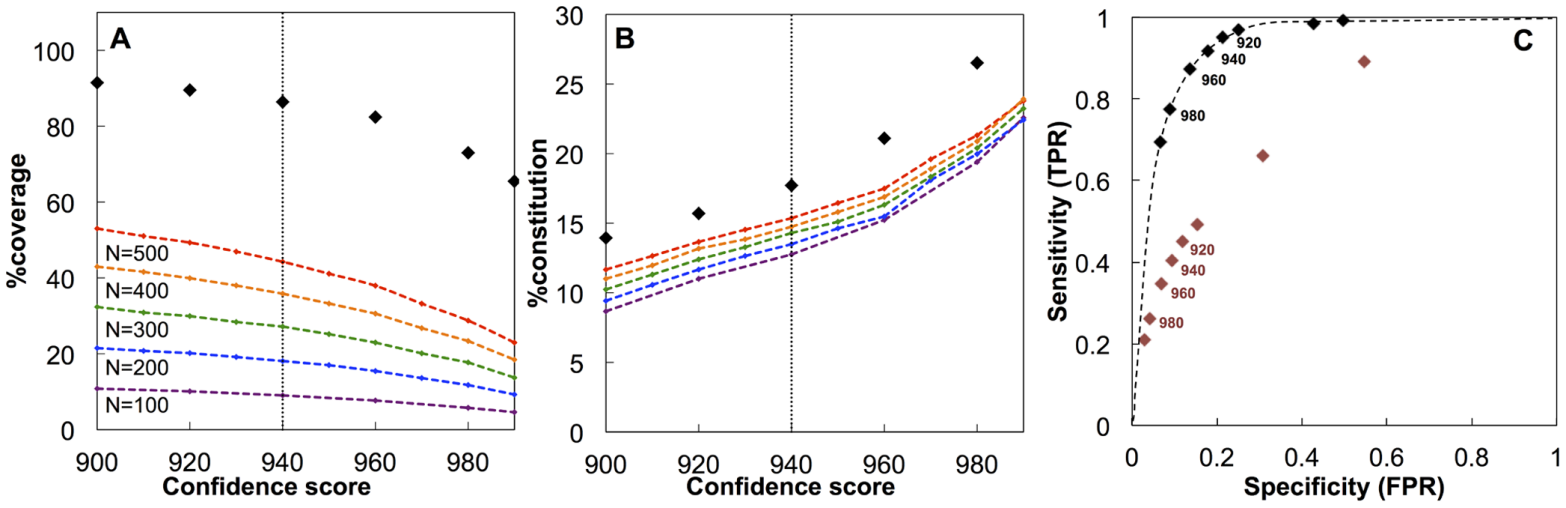
The (a) coverage, (b) constitution of the core proteins with respect to confidence scores. *N* shows the number of randomly selected proteins as the core proteins to construct the network. (c) ROC curve showing the trade-off between sensitivity and specificity for choosing the confidence score for interactions, red diamonds represent randomly generated networks.

Figure 3A displays the coverage of the network with respect to confidence score and Figure 3B shows how the number of proteins changes with increasing confidence score. These relations were also compared with 200 randomly generated networks, where 100, 200, 300, 400 and 500 randomly selected proteins were used to construct networks with corresponding confidence scores. The difference in the coverage and constitution measures between the disease specific and random networks indicates the coherency of the proteins. Since the initial 574 proteins (core proteins) are already associated with a disease (i.e. presumably functionally related), more core proteins are captured in the final network representation. For instance, for a set of 500 randomly selected proteins, the maximum achievable coverage at 900 is 53.02%, whereas the coverage of the network constructed by disease specific core proteins is 91.2%, indicative of biological relatedness. In this resulting network, however, at 900 confidence score, the constitution of the core proteins in the network is 13.37% (i.e. 13.7% of the final network representation is core proteins). Although, 91.2% of core proteins (c-proteins) were captured at the confidence score 900, such low confidence score leads to the presence of many redundant proteins. We observed a slight decrease in the coverage of the core proteins at 940; in return the constitution of the core proteins was increased up to 17.71%, indicating the elimination of the redundant neighboring proteins. Therefore, confidence score of 940 was accepted as the threshold to eliminate the linkages while keeping the sufficient amount of core proteins in the network. The sensitivity and specificity of the tested confidence scores were determined by calculating true-positive and false-positive rates (Figure 3C), where the former is the number of the core proteins in the final network representation; the latter is the non-core proteins. When 574 core proteins were extended with all possible neighboring proteins (i.e. without confidence score restriction), the final network representation contains 13488 proteins, which is the maximum size attainable with this core set of proteins. These values are compared with 200 randomly generated networks with 574 nodes, indicated with red diamond markers.

Following the removal of singletons, the giant component of network has 2770 nodes (proteins) and 15041 edges (functional linkages) and entitled as Type 2 diabetes related functional linkage network (T2DFN) (Table S5) and topological properties of the network were investigated as described previously (Text S1). [9] In this network, among the 2734 proteins, 497 of them are c-proteins, which have previously defined associations with the disease. These 497 proteins form 17.71% of the functional linkage network.

### Enumeration of Functional Modules in T2DFN

Genes participate in similar biological processes, share GO terms and operate in similar functions have a tendency to localize as dense groups in interaction networks [14]. These entities are considered as functional modules, where the members functionally linked to each other. The functional modules in T2DFN were derived based on modularity measure, as explained in Methodology. The Python scripting language is implemented to decipher the functional modules, where the members have the maximum allowable interaction, hence *Q* = 1. The algorithm used in this study, rather than assigning proteins into distinct clusters, allows the presence of proteins in many functional modules. The algorithm produced 10109 functional modules, the size of the modules ranges from two to 14, with an average module size of 4.04, hence with the supporting information that modules consisting four or more members are biologically meaningful [69], the 5414 modules of size four and above were considered for further analyses (Table S5).

### Evaluation and Scoring of T2DFN Modules

The module enumeration algorithms produce massive amounts of entities that require an elaborate analysis to elucidate the most informative and reliable components. There is accumulating evidence that proteins function together to exhibit a single action often tend to participate in similar pathways, co-localized and share GO Terms. In fact, genes contributing to a disorder have increased tendency for their products to be functionally related. Hence, the functional modules enumerated from T2DFN were evaluated and scored in terms of participation in pathways, co-localization and association with similar diseases. Combination of various resources provided a deliberate and consistent evaluation method.

The scoring of the functional modules was initiated with the assembly of the data that will be incorporated. KEGG Pathway database [64] was used to associate biological pathways, where the classification scheme involves 338 pathways, including major metabolic processes, as well as disease pathways. LOCATE database [65] was used to determine the co-localization information, where the proteins are assigned to 30 different subcellular compartments. In order to establish links between proteins and diseases, in this study, Medical Subject Headings (MeSH) [66] were incorporated with OMIM database to achieve the disease associations. Manual curation of the MeSH database yielded 3630 disease terms and these disease terms were categorized into 23 different disease classes depending on the system that is exposed to disease. These 3630 disease terms were then searched in OMIM database with an in-house developed text-mining algorithm to associate genetic information with the diseases. To assess the reliability of the scores assigned to the modules, the associations in the classification schemes were randomly shuffled 10^3^ times and calculated score was compared with the distribution of the random scores. After scoring of the functional modules was completed, three different scores were obtained: *R_KEGG_*, *R_LOC_* and *R_OMIM_*. These scores were varied from zero to one, where one indicates the consistency in the module. The selection of the most informative functional modules was completed using a non-linear model, where the parameters were estimated using genetic algorithm (GA).

### Construction of Condensed T2DFN

According to the model parameters estimated by GA, the functional modules were ranked according to their scores. The proteins in top scoring (25, 50, 75, 100, 150 and 250) functional modules were assembled. Each tentative condensed network was then analyzed in terms of the number of core proteins captured and the number of distinct sub-networks observed. The number of core proteins in this condensed network indicates the effect of the reduction in the network. The number of sub-networks formed accounts for the conjunction of the biological processes in the disease. In fact, the presence of many distinct sub-networks in a condensed network indicate diverse biological functions, however, does not provide information on how these biological processes are connected to each other. The proteins collected in a condensed map were anticipated to represent the fundamental biological processes involved in Type 2 diabetes.

The selection criterion for the number of top scoring functional modules that will be assembled was based on the number of core proteins and clusters. Since, the motivation of such assemble is to reveal underlying shared mechanisms; the number of clusters obtained through incorporation of the overlapping members of the selected functional modules is an important parameter. In addition, the number of core proteins in the network is another issue that should be considered to preserve the association with the disease. Considering the increase in the number of core proteins captured in the final network representation (which saturates at 26%), the top scoring 75 functional modules were incorporated to construct a condensed functional linkage network. Selection of these modules is explained in Text S1.

**Figure 4:**
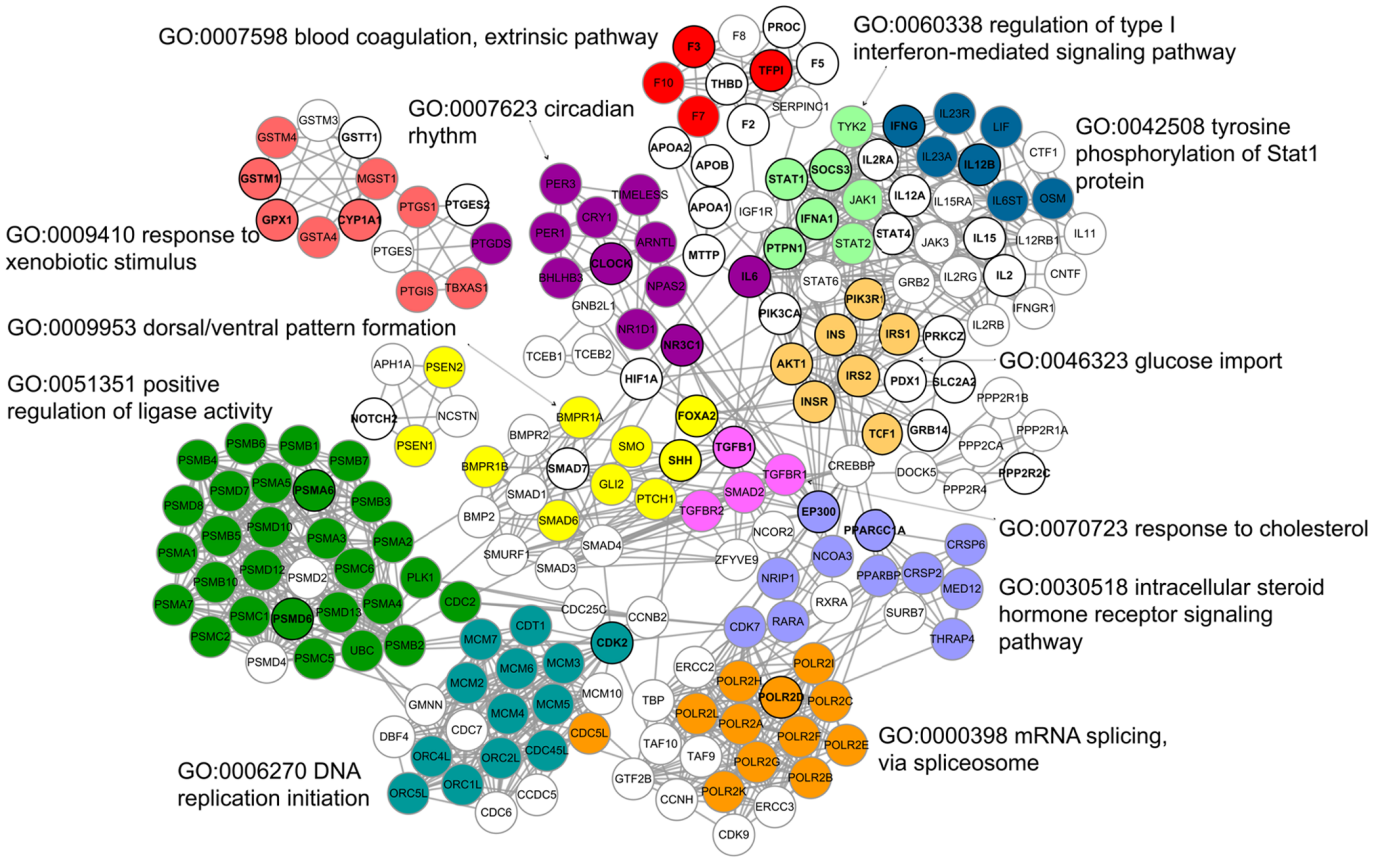
Condensed functional linkage network constructed from the top scoring functional modules in T2DFN. In this representation, white color represents the proteins that are not associated with a GO Term in the final configuration.

Among 5414 modules, the overlapping members of 75 top scoring functional modules were assembled reflecting the biological processes involved in the disease. This condensed network, presented in Figure 4, contains 203 proteins, 149 of which are non-core proteins, and 1118 interactions (Table S5). The core proteins in the assembly (Figure 4) are indicated with black borders.

### Indicators of Fundamental Cellular Processes in T2DFN

To elucidate the fundamental biological processes involved in the disease, The Gene Ontology terms for the condensed network proteins were extracted. These biological process terms contain excessive amount of overlaps among proteins impeding a comprehensive analysis. (Table S3) A novel computational framework explained in Methods was followed to decipher the underlying distinct biological processes in the condensed network. This computational procedure resulted in over 50,000 possible combinations of GO Terms. The best configuration of GO Terms was selected to achieve a comprehensive representation of the network (Figure 4). As a result, 12 GO Terms with non-overlapping 124 members indicated the major cellular processes involved in the progression of the disease. The GO Terms enriched in this condensed map is represented in the Table 1. In Figure 4, the nodes are colored according to the distinct GO Term. In this representation, white color represents the proteins that are not associated with a GO Term in the final configuration. However, this non-association does not necessarily imply that a particular protein is not associated with a biological process; rather it is an implication of its involvement in many biological processes.

**Table 1:** 12 distinct GO terms corresponding to separate cellular processes enriched in the T2DFN condensed network.

GO Term	p-value	Proteins
GO:0051351 positive regulation of ligase activity	5.44E-52	PLK1, PSMD13, UBC, PSMD6*, PSMC5, PSMC6, PSMB4, PSMB2, PSMA3, PSMC1, PSMD10, PSMA5, PSMB1, PSMA6*, PSMC2, PSMB6, PSMA7, PSMA1, PSMB7, PSMB3, PSMA4, PSMA2, PSMD7, PSMD12, PSMD8, PSMB5, PSMB10, CDC2
GO:0006270 DNA replication initiation	1.78E-17	ORC1L, MCM5, CDC45L, CDK2*, MCM6, MCM7, ORC5L, ORC2L, MCM2, ORC4L, MCM3, CDT1, MCM4
GO:0007623 circadian rhythm	2.87E-13	PER1, CRY1, NR3C1*, BHLHB3, CLOCK*, PTGDS, ARNTL, NPAS2, PER3, NR1D1, IL6*, TIMELESS
GO:0060338 regulation of type I interferon-mediated signaling pathway	4.73E-10	SOCS3*, TYK2, STAT2, JAK1, PTPN1*, STAT1*, IFNA1*
GO:0030518 intracellular steroid hormone receptor signaling pathway	2.05E-09	MED12, RARA, EP300*, CRSP6, THRAP4, CDK7, CRSP2, NRIP1, PPARGC1A*, NCOA3, PPARBP
GO:0042508 tyrosine phosphorylation of Stat1 protein	2.11E-09	IL23R, IL23A, IFNG*, LIF, OSM, IL6ST, IL12B*
GO:0000398 mRNA splicing, via spliceosome	1.02E-08	POLR2C, POLR2B, POLR2L, CDC5L, POLR2D*, POLR2I, POLR2H, POLR2F, POLR2E, POLR2A, POLR2K, POLR2G
GO:0009953 dorsal/ventral pattern formation	1.68E-08	SHH*, SMO, FOXA2*, SMAD6, BMPR1A, PSEN2, PSEN1, BMPR1B, GLI2, PTCH1
GO:0007598 blood coagulation, extrinsic pathway	2.72E-08	F10, F3*, TFPI*, F7
GO:0009410 response to xenobiotic stimulus	3.19E-07	PTGS1, PTGIS, CYP1A1*, TBXAS1, GPX1*, GSTM1*, MGST1, GSTA4, GSTM4
GO:0070723 response to cholesterol	3.74E-06	TGFBR2, TGFBR1, SMAD2, TGFB1*
GO:0046323 glucose import	8.92E-06	IRS1*, IRS2*, INSR*, TCF1*, AKT1*, INS*, PIK3R1*

* core proteins

Dorsal/ventral pattern formation term (*p-val* = 1.68E-08) consists of signal transducer and transcriptional modulator (SMAD6), forkhead class of DNA-binding protein (FOXA2), smoothened homolog (SMO), sonic hedgehog homolog (SHH), patched homolog 1 (PTCH1), glioma-associated oncogene family zinc finger 2 (GLI2), presenilin-1 (PSEN1), presenilin-2 (PSEN2) and bone morphogenetic protein receptors (BMPR1A and BMPR1B) proteins and display the modulating function of Hedgehog (Hh) signaling in diabetes. Hedgehog signaling is known to function in early pancreas development, [70] and defects are considered as a potential factor in T2D. [71].

Positive regulation of ligase activity (*p-val* = 5.44E-52) was found to be one of the distinct GO process terms, which is involved in the pathogenesis of T2D in the present study. The cluster, which is significantly associated with this process, consists of 28 proteins including all the subunits of 20S core and 19S regulator particles of 26S proteasome. Two members of this cluster, PSMA6, and PSMD6, have already reported associations with Type 2 diabetes (core proteins). PSMA6 gene encodes a member of 20S core α-subunit type 6, PSMD6 encodes a non-ATPase subunit of the 26S regulator. PSMD6 is probably involved in the ATP-dependent degradation of ubiquitinated proteins [72]. Although the mechanism leading to β-cell dysfunction causing T2D is not well understood, there is accumulating evidence that it is related to β-cell endoplasmic reticulum (ER) stress, increased β-cell apoptosis. The ER functions as a quality control system to target the unfolded proteins to the ubiquitin-proteasome system (UPS). The up-regulation of the UPS in rat muscle with T2D was also reported [73]. The investigation of the myocardial infarction susceptibility in Type 2 diabetes showed that UPS plays also an important role in arterial plaque formation [74], hence the up-regulation of the UPS was suggested to be potential mechanism that links myocardial infarction to Type 2 diabetes [75]. UPS is activated by various stimuli, including oxidative stress and plays a pivotal role in the activation of nuclear factor B (NF-κB) transcription factor, which induces the transcription of proinflammatory cytokines [74].

Response to cholesterol (*p-val* = 3.74E-06) was found to be distinctly and significantly associated with the cluster including TGFB1, TGFBR2, TGFBR1 and SMAD2, where only TGFB1 is a core protein. Transforming growth factor β1 (TGFB1) is a ubiquitously expressed in humans, its levels are up-regulated in some cancers, and play important physiological roles in tissue regeneration, cell differentiation, embryonic development, the regulation of the immune system and apoptosis [76]. It is known that hyperglycemia is one of the major factors for TGFB1 expression, and patients with diabetes have higher levels of TGFB1 than healthy people. TGFB1 induces the phosphorylation of the TGF-β receptor activated protein (SMAD2), and its responsiveness is modulated by cholesterol by binding TGFB receptors [77]. SMAD2 transfers the signal of the TGFB, and regulates cell proliferation, apoptosis, and differentiation. The interaction of SMAD2 with double zinc finger FYVE domain protein (ZFYVE9) enables SMAD2 to be recruited to TGFB receptors. Followed by TGFB signal, this complex is dissociated and SMAD2 forms a complex with SMAD4. The association enables SMAD2 to be directed to the nucleus, where it binds to target promoters and forms a transcription repressor complex with other cofactors. SMAD2 can also be phosphorylated by activin type 1 receptor kinase, and mediates the signal from the activin. Activin signaling pathway has recently suggested as a potential therapeutic target for obesity associated metabolic complications [78]. The existence of SMAD proteins with TGFB signaling and proteasome unit members suggests that SMAD proteins might have regulatory roles in the proteasome activity through activin signaling, leading to the suppression of PI3K signaling and decreased insulin expression. This functional module was previously associated with kidney failure in diabetes in network study integrating gene-gene co-expression with protein interaction data. [13].

Type 1 Interferon-mediated signaling pathway (*p-val* = 4.73E-10), which can be considered as a part of the JAK-STAT pathway, is represented with STAT1, STAT2, SOCS3, IFNA1, PTPN1, JAK1, TYK2, where four of them have well characterized roles in the pathophysiological processes of the disease. JAK-STAT signaling pathway transmits extracellular signals from a variety of cytokines, lymphokines and growth factors to the nucleus and its activation stimulates cell proliferation, differentiation, migration and apoptosis. It has also been reported that high glucose concentrations induces the production of TGFB and activates JAK-STAT cascade [13]. Two STAT proteins (STAT1 and STAT2) out of seven members were captured within this large cluster. SOCS3 is a member of suppressors of cytokine signaling (SOCS) proteins, which are also known as JAK-binding protein. The members of this family of proteins are responsible for establishing inducible negative regulations of cytokine signaling via inhibition of JAK-STAT pathway. Cytokine-induced activation by STATs is a major mechanism of SOCS induction; however, there is increasing evidence that SOCS expression can also be induced by other stimuli, such as elevated levels of lipopolysaccharide and insulin [79]. SOCS3, which is a major suppressor of JAK-STAT signaling, is reported to inhibit JAK1 and TYK2 [80]. In a study aiming to understand the interplay between cardiovascular disease and other complex disorders, Rende et al have identified SOCS3 in a functional module consisting of INSR, INS, IRS1 and LEP and found to be significantly linked with Diabetes Mellitus, hypertriglyceridemia and hypoglycemia. The presence of SOCS3 in this module was attributed to the formation of a link between cardiovascular disease and diabetes [9].

12 proteins formed a cluster that is enriched with circadian rhythm GO biological process term (*p-val* = 2.87E-13). Transcriptional activator of the molecular clock consists of a heterodimer between either the CLOCK or the neuronal PAS domain protein 2 (NPAS2) and the aryl hydrocarbon receptor nuclear translocator-like protein (ARNTL) that binds to E-box elements in the promoter of three period (PER) and two cryptochrome (CRY1) genes, thereby activating their transcription [81]. The association of CRY1 and NPAS2 single nucleotide polymorphisms with the disease has been reported in a recent study focused on exploiting the association of type 2 disease and circadian rhythm genes. [82] A number of other genes, such as nuclear receptor subfamily 1, group D, member 1 (NR1D1), and timeless homolog (Drosophila) (TIMELESS), are involved in the feedback loops. Type 2 diabetes is associated with increased incidence of hypertension and disrupted blood pressure (BP) circadian rhythm [83] and people having rotating night shifts are susceptible to the disease, partly mediated through body weight [84]. These results show the indicative role of circadian rhythm genes Type 2 diabetes susceptibility. Our results showing the involvement of these proteins in the condensed network also suggest that successful maintenance of circadian rhythm is an important parameter that needs to be controlled during the progression of the disease.

11 proteins (CDK7, CRSP2, CRSP6, EP300, NRIP1, NCOA3, RARA, PPARBP, PPARGC1A, MED17, THRAP4) are enriched with steroid hormone receptor signaling pathway (*p-val* = 9.39E-04), where PPARGC1A and EP300 are core proteins. NRIP1, NCOA3, RARA are nuclear receptors that translate hormonal, metabolic and nutritional signals into various metabolic activities by altering gene expression. Nuclear receptor interacting protein 1 (NRIP1) modulates transcriptional activity of the estrogen receptor by steroid receptors. NRIP1 was shown to act either as a transcriptional repressor or activator depending on the transcriptional factors with which it interacts. This finding underlines its essential role in normal cellular function and metabolic diseases [85]. Retinoic acid (RA), which is biologically active metabolite of vitamin A (retinol), plays an essential role in embryonic eye development and maintains vital organs in adults [86], [87]. Vitamin A metabolism is strictly controlled by various retinoid-generating enzymes, retinoid-binding proteins and retinoid-activated nuclear receptors. Retinoic acid receptor (RARA) has also regulatory roles in regulation of development, differentiation, apoptosis, transcription of clock genes [88]. The protein encoded by the gene PPARGC1A is a transcriptional regulator that is involved in energy metabolism and is an important factor regulating the expression of genes for oxidative phosphorylation and ATP production in target tissues through co-activation of nuclear receptors. PPARGC1A mRNA expression has been found to be correlated with glucose-stimulated insulin release, and its inhibition of expression was shown to be associated with a decline in INS mRNA expression [89]. PPARBP (MED1), CRSP2 (MED14), CRSP6 (MED17) and THRAP4 (MED24) are the components of the mediator complex, which is involved in the regulation of hormone receptor–dependent transcription of selected genes by acting as a bridge between transcription factors and RNA polymerase II. [90], [91] None of the components of the mediator complex captured in this study were reported to be associated with the disease. The assembly of these proteins under steroid hormone receptor signaling process and the presence of nuclear receptors in T2DFN suggest that along with their diverse actions in sterol, retinoic acid, thyroid and glucocorticoid metabolism; nuclear receptors portray prospective therapeutic targets in regulating these metabolic processes.

### Disease Interventions Derived from T2DFN

**Figure 5:**
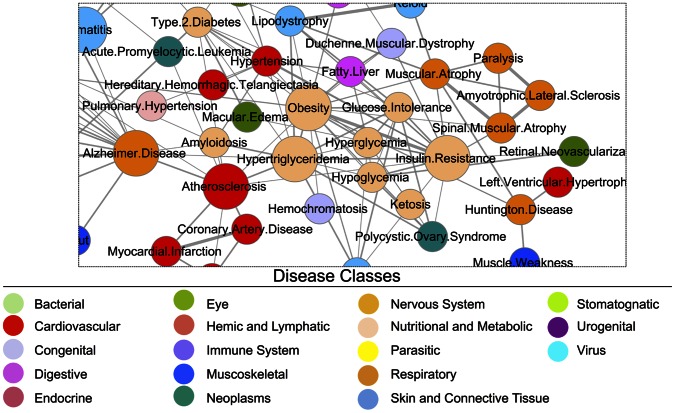
A section of disease network showing disease associations derived through T2DFN.

The proteins involved in the modular form in T2DFN have been implicated to be present in many other complex diseases; therefore the diseases can be linked to each other through shared proteins. To elucidate the shared partners, the proteins in condensed form of T2DFN were used to calculate the disease overlapping score among diseases. The manual curation and elimination of the MeSH terms initially yielded 3630 disease terms, and subsequently incorporated with OMIM database records, forming a disease classification scheme, which was also used scoring the functional modules enumerated from T2DFN (Table S4). The 203 proteins present in condensed map were related with 370 disease terms. According to the shared proteins, the disease overlapping score was calculated for each pair of diseases, and significantly associated 146 diseases sharing at least two proteins were selected (*p-val*<1.00E-02). A representative network showing Type 2 diabetes and its related neuromuscular diseases are presented in Figure 5 (The entire disease network is presented in Figure S3, all pairwise disease relations were given in Table S6). In this network, the nodes are colored according to disease class and node sizes adjusted according to the number of links established with other disease.

**Figure 6:**
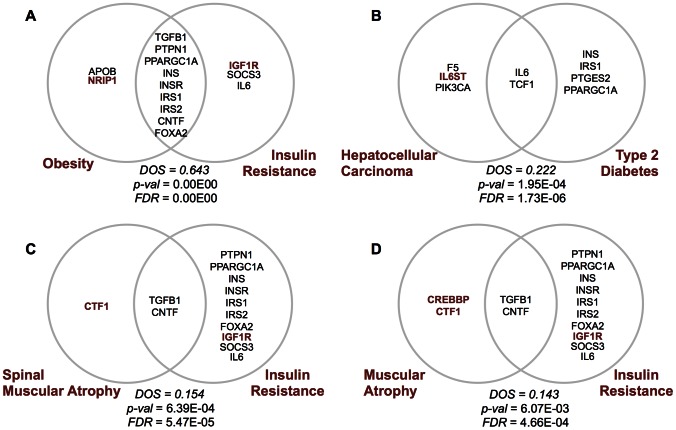
A representative scheme showing the links between selected metabolic and neuromuscular disorders derived from the protein linkages in T2DFN.

The relation between insulin resistance and obesity is established through nine shared proteins, all of which are core proteins (Figure 6A), presented at the intersection, and the proteins that are not present in the core network are indicated with red. In this scheme, the non-core proteins, namely, NRIP1, is linked with obesity; IGF1R is linked with insulin resistance. Nuclear receptor interacting protein 1 (NRIP1), also known as RIP140, is a nuclear protein that specifically interacts with the hormone-dependent activation domain of nuclear receptors, such as estrogen receptor. RIP140 suppresses the expression of gene clusters that are involved in lipid and carbohydrate metabolism, inhibits glucose uptake and facilitates the expression of genes promoting energy expenditure. Therefore, the functional interplay between transcriptional activators and RIP140 is an essential process in metabolic regulation [92]. However, two recent studies reported contradictory results; the function and expression level of RIP140 was not correlated with obesity [93] but lower gene and protein expression levels of RIP140 was observed in obese subjects [94]. Although controversial studies were reported on the modulating effect on RIP140 in obesity, RIP140 has an obvious modulating role in lipid and carbohydrate metabolism, and one recent study reported the role of RIP140 in maintaining energy homeostasis and a promising therapeutic target for insulin resistance [95].

One other relation derived from disease overlapping network is hepatocellular carcinoma (HCC) and Type 2 diabetes (Figure 6B), where IL6 and TCF1 are shared. Hepatocellular carcinoma is the most common form of liver cancer. The mutations on IL6ST are associated with hepatocellular carcinoma. [96] In a recent study, the incidence of HCC is reported to be as twice higher for diabetes patients. [97].

The associations between insulin resistance, spinal muscular atrophy (SMA) and muscular atrophy are presented (Figure 6C and 6D), where ciliary neurotrophic factor (CNTF) and transforming growth factor beta 1 (TGFB1) are shared. Muscular atrophy is the loss of mass and strength that progresses with medical conditions such as cancer and aging. Spinal muscular atrophy, on the other hand is a neuromuscular disease that is characterized by degeneration of motor neurons, leading to progressive muscular atrophy. Cardiotrophin-1 (CTF1) is a muscle-derived member of IL6 family cytokine, exerts its cellular effects by interacting with the glycoprotein 130 [98], and is highly expressed in embryonic skeletal muscle and secreted by myotubes [99]. It promotes the survival of cultured embryonic mouse and rat motor neurons. Circulating levels of CTF1 were associated with glucose levels, where glucose triggers CTF1 expression in adipocytes [98]. In SMA, CTF1 has a slowing down effect on the progression of the disease [100]. This finding suggests that CTF1 has a modulating effect on the metabolic processes involved in diabetes and neuromuscular diseases. CREB binding protein (CREBBP) is associated with muscular atrophy, which is ubiquitously expressed and is involved in the transcriptional co-activation of many different transcription factors. CREBBP has also been implicated to play a central role in spinal and bulbar muscular atrophy, which is a neurodegenerative disorder caused by toxic effects of polyglutamine tracts [101]. In animal models, heterozygous CREBBP deficiency results in increased effects of hormones such as adiponectin and leptin, preventing obesity and insulin resistance. Hence, CREBBP functions as a “master-switch” between energy storage and expenditure through inhibition or activation of leptin and adiponectin pathways [102].

### Conclusions

In the present study, an integrative modular approach based on the functional linkage network associated with Type 2 diabetes was developed to investigate statistically significant metabolic processes in the disease. The proteins clustered in the functional modules were scored and evaluated in terms of shared pathways, co-localization, and co-occurrence with other complex diseases. The assembly of top scoring overlapping members in the functional modules revealed the fundamental biological processes present in the pathophysiology of the disease. It should be noted that the prospect of inferring biological information from networks that are constructed by publicly available databases contains biased information to a certain extent, the set of known interactions are overrepresented and less studied interactions have less evidence to support. The interaction databases are biased toward proteins from particular cellular components and processes conserved proteins and highly expressed proteins.

Along with glucose and cholesterol related processes, the proteins related to circadian rhythm appeared as a cluster in the condensed network, which underlines the fact that successful maintenance of circadian rhythm is an important parameter that needs to be controlled during the progression of the disease. The nuclear receptors, which have diverse actions in sterol, retinoic acid, thyroid and glucocorticoid metabolism, also appear in the condensed network, indicative of possible roles in the disease.

In the current study, the disease’s association with other complex disorders was established through shared proteins. The statistically significant overlap between the diseases indicated that the protein, ciliary neurotrophic factor, encoded by CNTF might have a modulating role in linking obesity, insulin resistance and neuromuscular diseases, namely spinal muscular atrophy. Nuclear receptor interacting protein 1 (NRIP1), which is not previously associated with Type 2 diabetes, links insulin resistance with obesity. NRIP1 has a modulating role in lipid and carbohydrate metabolism, studies were yet to be conducted to investigate the subsequent effects of NRIP1 on Type 2 diabetes. Our results showed that CREB binding protein (CREBBP), which has roles in adiponectin, leptin signaling pathways and energy storage, links muscular atrophy to insulin resistance. Our study also revealed that Cardiotrophin-1 (CTF1) is present in the conjunction of insulin resistance and spinal muscular atrophy. Although it is not previously associated with Type 2 diabetes, evidence indicating that circulating levels of CTF1 are associated with glucose levels suggests its role in linking Type 2 diabetes and neuromuscular diseases.

## Supporting Information

Figure S1 **(a) Degree,** ***n*** **(** ***k*** **), and (b) average clustering coefficient, <** ***C*** **(** ***k*** **)>, distribution of T2DFN with respect to degree,** ***k*** **, distribution in T2DFN.**
(TIF)

Figure S2 **Distribution of functional module scores.**
(TIF)

Figure S3 **Entire disease-disease association network.**
(TIF)

Table S1 **Estimated nonlinear model parameters for T2DFN.**
(XLSX)

Table S2 **Selection criteria of the top scoring functional modules in T2DFN according to the results obtained from Genetic Algorithm.** *N* and *l* are the number of proteins and the linkages in the condensed network, respectively. C-proteins are the core proteins, which are already associated with Type 2 diabetes. Distinct subnetworks indicate the number of distinctive groups observed in the condensed network.
(XLSX)

Table S3 **All GO Terms associated with the network.**
(XLSX)

Table S4 **Curated data set used to associate genes to diseases.**
(XLSX)

Table S5 **Core set of proteins, functional linkage network, functional modules enumerated, condensed T2D network.**
(XLSX)

Table S6 **Pairwise disease associations.**
(XLSX)

Text S1 **Network Construction, Characteristics; Scoring and Selecting Functional Modules.**
(DOCX)
