# Supplementary figures and images for: Complex Disease Interventions from a Network Model for Type 2 Diabetes
Source: PLoS One. 2013 Jun 11;8(6):e65854. doi: 10.1371/journal.pone.0065854 (PMC3679160; doi:10.1371/journal.pone.0065854)

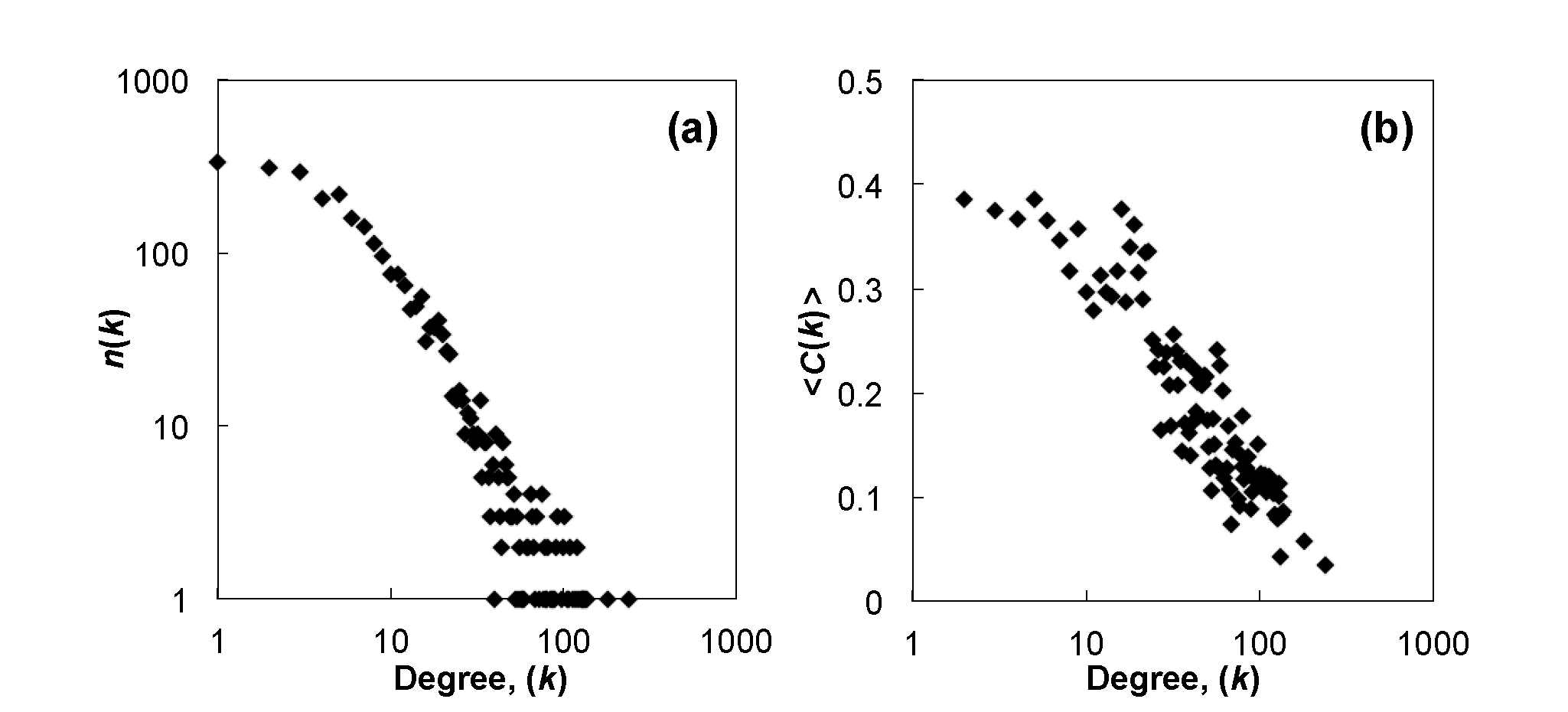

Supplement: Figure S1 — (a) Degree, n ( k ), and (b) average clustering coefficient, < C ( k )>, distribution of T2DFN with respect to degree, k , distribution in T2DFN. (TIF) [file pone.0065854.s001.tif]

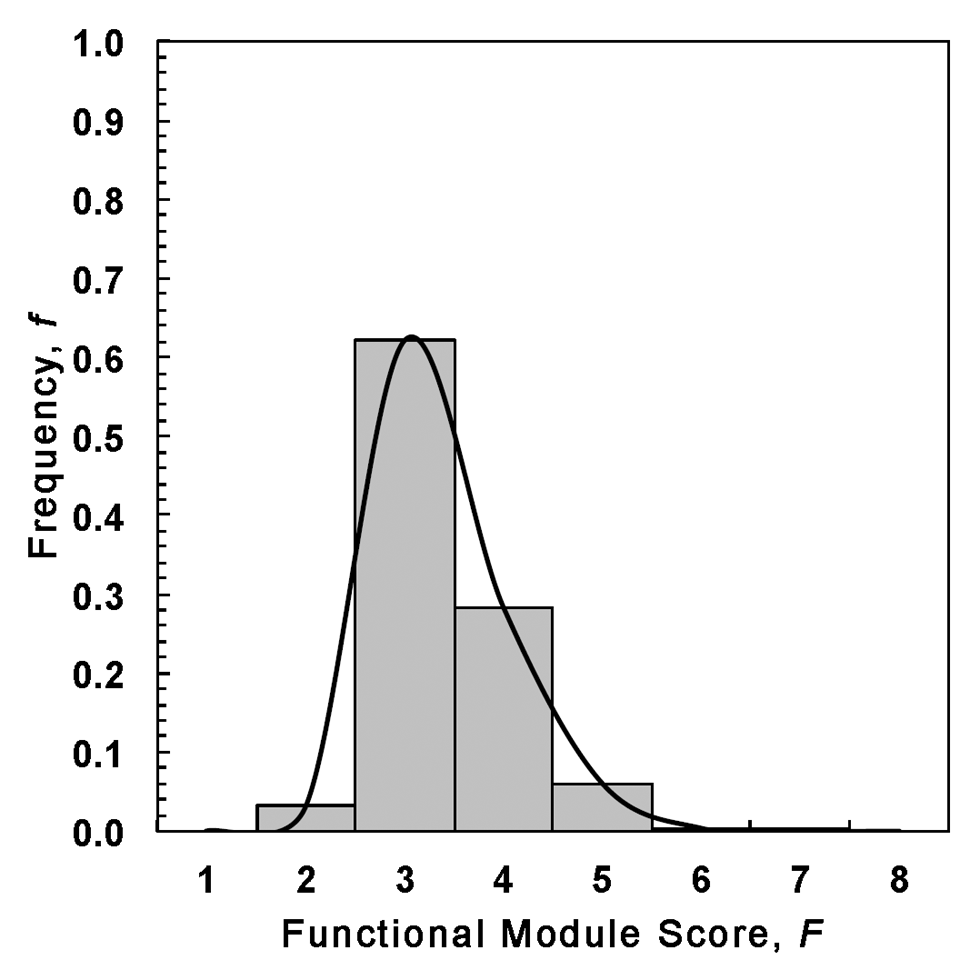

Supplement: Figure S2 — Distribution of functional module scores. (TIF) [file pone.0065854.s002.tif]

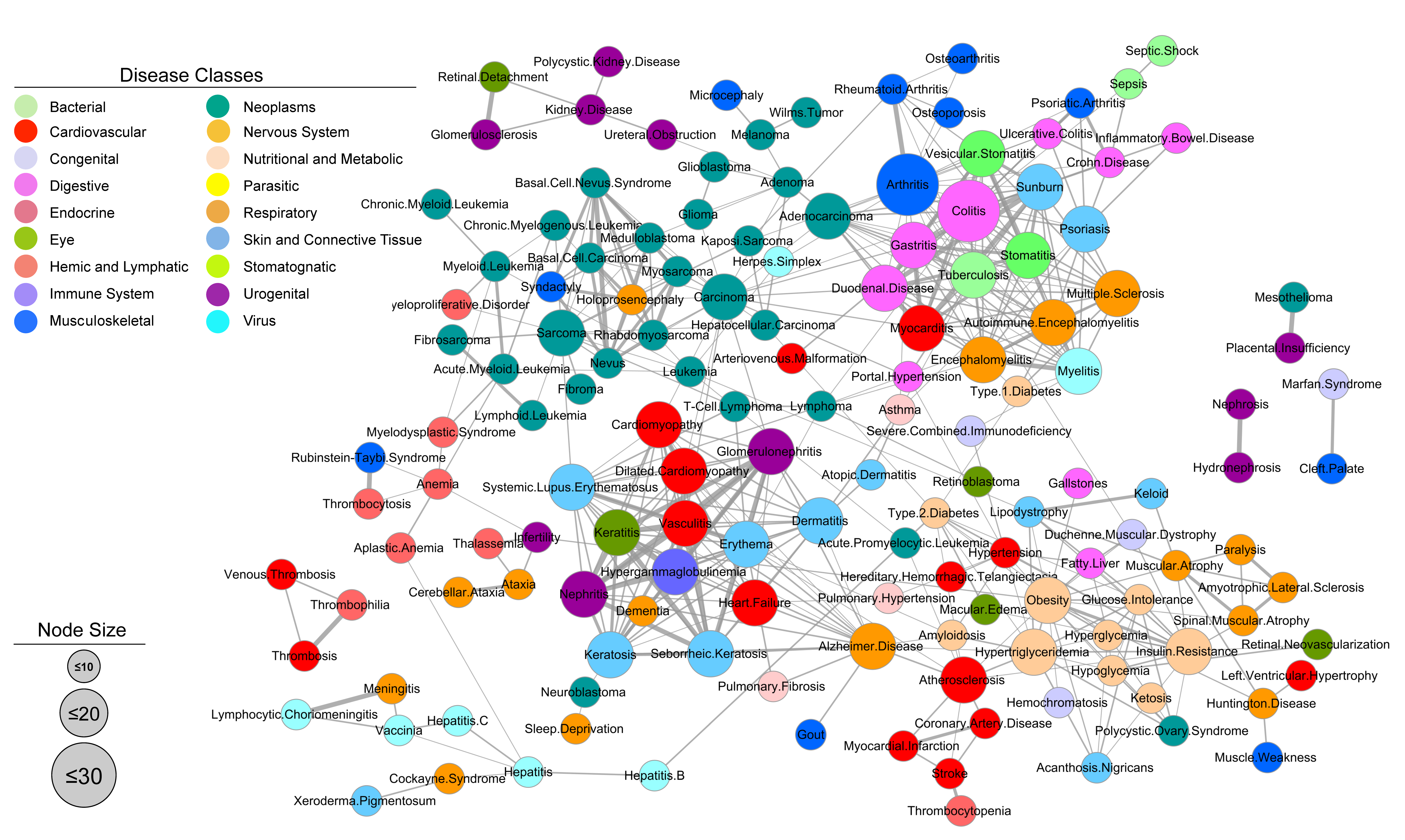

Supplement: Figure S3 — Entire disease-disease association network. (TIF) [file pone.0065854.s003.tif]
