# Supplementary material for: Complex Disease Interventions from a Network Model for Type 2 Diabetes
Source: PLoS One. 2013 Jun 11;8(6):e65854. doi: 10.1371/journal.pone.0065854 (PMC3679160; doi:10.1371/journal.pone.0065854)
Supplement: Text S1 — Network Construction, Characteristics; Scoring and Selecting Functional Modules. (DOCX) [file pone.0065854.s010.docx]

Complex Disease Interventions from a Network Model for Type 2 Diabetes

**Text S1. Network Construction, Characteristics; Scoring and Selecting Functional Modules**

**Network Construction and Characteristics**

The disease related network construction started with an initial set of proteins, named as core-proteins, which have reported associations with the disease. To capture other putative proteins that have potential links with a disease, the first neighbors of the core proteins were also extracted from STRING database. All of the proteins that interact with the core proteins, regardless of the confidence score, were accepted as “candidate neighbors”. Among these candidates, only the ones with a qualified interaction (having a confidence score > threshold value) were included in the network. The nodes that are not connected to the giant component and their interconnections were excluded, and finally the construction of the network of interest was completed.

To select the confidence threshold for functional linkages to achieve a comprehensive representation of the system under investigation, various disease-related functional linkage networks were constructed with changing confidence score for interactions. Starting from a lenient criterion (900) to a stringent score (990), each network was analyzed in terms of two measures: coverage and constitution. Coverage measure is defined as the fraction of number of core proteins included in the network to the number of proteins initially collected. Constitution measure was also defined as the number of core proteins included in the network to the number of total proteins. The aim was to keep maximum number of core proteins in the network, while considering the fraction of the number of core proteins in the network. Upon the selection of a suitable confidence score, the nodes that are not connected to the giant component of the network were eliminated for computational purposes.

For Type 2 diabetes functional linkage network (T2DFN), the average degree (<*k>*), average clustering coefficient (<*C_i_*>) distributions were calculated by Network Analyzer, a plugin for Cytoscape [[1](#_ENREF_1)]. The interaction networks are defined as sets of *N* nodes, representing the proteins and *l* edges, representing the interactions among them.

(i) Degree (*k*) is the number of the interactions that one node has [[2](#_ENREF_2)]. The average degree <*k*> for a network is defined as:

|  | $\left\langle k\right\rangle=\frac{2l}{N}$ | (S1) |
| --- | --- | --- |

(ii) Clustering coefficient (*C_i_*) is the fraction of the number of existing interactions among the neighbors of a particular node, *l_i_*, to the maximum allowable interactions among them. *C_i_* ranges from 0 to 1, where 0 indicates that the neighbors of a particular node are not connected. This measure provides information on how the neighbors are interconnected [[2](#_ENREF_2)]. The average clustering coefficient is:

|  | $\left\langle C\right\rangle=\frac{1}{N}\sum_{i=n}^{N} \frac{2l_{i}}{k_{i}(k_{i}-1)}$ | (S2) |
| --- | --- | --- |

Real biological networks exhibit a scale-free behavior, indicating that many proteins have low number of interactions, whereas fewer proteins have higher degrees of interaction. It is known that the degree distribution for a scale-free network follows the Power law, i.e. *f* (*k*) = *Ak^-γ^,* where *f* (*k*) is the frequency of nodes, 2 < *γ* < 3 and *A* is a constant. The topological features of T2DFN were analyzed in terms of the degree, *k*, and clustering coefficient, *C,* for each node. The average degree <*k*>, and average clustering coefficient <*C*> for the network were calculated to be 10.86, 0.293 respectively. The degree distribution *n*(*k*) (Figure S1(a)) and clustering coefficient distribution *C*(*k*) (Figure S1(b)) with respect to degree distribution, followed the Power law, indicating that many proteins are linked to a few other proteins but only a few of them have many interactions, hence providing additional evidence that the network of interest exhibits a scale-free behavior with a degree exponent, *γ* = 2.588 (R^2^ = 0.888).

**Genetic Algorithm**

After scoring of the functional modules was completed, three different scores were obtained: *R_KEGG_*, *R_LOC_* and *R_OMIM_*. These scores were varied from zero to one, where one indicates the consistency in the module. The most informative functional modules were selected by a non-linear model that was proposed to evaluate the functional modules with a single resulting score.

|  | $F_{i}=\sum_{j} \alpha_{j}S_{j}^{\beta_{j}}$ | (S3) |
| --- | --- | --- |

where *S_j_* ∈ (log*N*, *R_KEGG_*, *R_LOC_*, *R_OMIM_*), all of which ranges between 0 and 1, 1 indicating consistency in the module; except for *N*, which denotes the size of the module. *α_j_* and *β_j_* are the nonlinear model coefficients.

Genetic Algorithm (GA) was employed to estimate the nonlinear model parameters. GA approach is a population based optimization technique that is designed to search optimum values in a complex space. The nonlinear model parameters were predicted by evolving the population of tentative solutions of the model in the search space. The ten artificially generated functional modules, five of which have the highest score in each scoring scheme, were planted in the population representing the best achievable entities. The population of the modules was evolved for 100 generations. The estimated model parameters were then used to evaluate the modules enumerated from T2DFN and summarized in Table S1.

According to these parameters, the functional modules were evaluated and the high scoring functional modules were investigated for biological significance. The functional modules’ score distribution is presented in Figure S2.

**Construction of Condensed Network By Selecting Top Scoring Functional Modules**

The motivation of condensing the initial network to a smaller network is to highlight the most informative proteins and biological processes in Type 2 diabetes. After the modules are scored, the highest scored modules are assembled. To identify shared proteins among these processes, the number of clusters obtained is the key point to decide the number of modules that is sufficiently enough to represent the biological processes. In addition, to preserve the network’s association with the disease, the number of core proteins were also taken into account. The procedure simply incorporates the top scoring 25, 50, 75, 100, 150 and 250 functional modules in different networks and control these networks in terms of the number of nodes, links, presence of core proteins and the number of distinct clusters. For instance, when top 25 modules are incorporated in a single network, the proteins are grouped in two clusters, however the number of core proteins in this network is low (the core proteins constitute 10.3% of the entire nodes). When top scoring 250 modules are used to construct a network, the number of clusters is four and the percentage of the core proteins in the entire network is 25.5%. When top scoring 75 modules are assembled, the clusters in the network is three and the core proteins are 26% of the entire network. The basis of the criteria is presented in the Table S2.

**References**

1. Assenov Y, Ramirez F, Schelhorn S, Lengauer T, Albrecht M (2008) Computing topological parameters of biological networks. Bioinformatics 24: 282 - 284.

2. Rodriguez-Caso C, Medina M, Solé R (2005) Topology, Tinkering and Evolution of The Human Transcription Factor Network. FEBS Journal 272: 6423-6434.
